# Does DNA Methylation Plays a Critical Role in Osteoblastic Differentiation of Mesenchymal Stem Cells (MSCs)?

**DOI:** 10.5812/ircmj.4615

**Published:** 2013-08-05

**Authors:** Najmaldin Saki, Majid Farshdousti Hagh, Esmaeil Mortaz, Abdolreza Ardeshiry Lajimi

**Affiliations:** 1 Research Center of Thalassemia and Hemoglobinopathy, Jundishapur University of Medical Sciences, Ahvaz, IR Iran; 2 Hematology and Oncology Research Center, Tabriz University of Medical Sciences, Tabriz, IR Iran; 3 Division of Pharmacology, Utrecht Institute for Pharmaceutical Sciences, Utrecht University, Utrecht, Netherlands; 4 Young Researchers Club, Science and Research Branch, Islamic Azad University, Tehran, IR Iran

**Keywords:** Mesenchymal Stromal Cells, Cell Differentiation, Osteoblastic

Dear Editor,

Mesenchymal stem cells (MSCs) are characterized by ability to differentiate into several cell types and self-renewability. These stem cells have a limited capacity in cell lineage differentiation including osteogenic, adipogenic, chondrogenic, or myogenic lineage differentiations (1). Regarding their ease of isolation and specific characteristics, MSCs have been used widely in regenerative medicine and tissue engineering (2). In spite of identification of several signaling molecules in MSCs differentiation, controlling mechanisms in MSCs differentiation has not well been described. Recently, epigenetic mechanisms have been identified as the master regulatory mechanism in MSCs differentiation such as DNA methylation, histone modification and regulatory micro RNAs (2-4). In this report, we investigate DNA methylation status of ROR2 gene in osteoblastic differentiation of MSCs. We also show that ROR2 promoter was hypomethylated during osteoblastic differentiation for which the details can be found in Noruzinia et al. (5); While other important osteoblastic specific genes did not evaluate. RUNX2 and OSX are two of the most known osteoblast specific transcription factors (6, 7). However, several other non-osteoblast specific transcription factors have been identified to control osteoblast differentiation, including TWIST1 (twist homolog 1), ZBTB16 (zinc finger and BTB domain containing 16), DLX5 and MSX2 (MSH homeobox homolog 2) (8, 9). Therefore, we suggest that those genes (such as RUNX2 and OSX) could be considered as a a subject for future investigations.

Besides, it can be postulated that osteoblastic differentiation of MSCs may be influenced by mechanisms other than DNA methylation (10). Therefore, we suggest that other epigenetic mechanisms including histone modification and regulatory micro RNAs regarded in osteoblastic differentiation of MSCs sould be considered for future studies.
